# Moderate-to-vigorous physically active academic lessons and academic engagement in children with and without a social disadvantage: a within subject experimental design

**DOI:** 10.1186/s12889-015-1745-y

**Published:** 2015-04-19

**Authors:** Marijke J Mullender-Wijnsma, Esther Hartman, Johannes W de Greeff, Roel J Bosker, Simone Doolaard, Chris Visscher

**Affiliations:** University of Groningen, University Medical Center Groningen, Center for Human Movement Sciences, Antonius Deusinglaan 1, Groningen, 9713 AV The Netherlands; University of Groningen, Groningen Institute for Educational Research, Grote Rozenstraat 3, Groningen, 9712 TG The Netherlands; University of Groningen, Faculty of Behavioral and Social Sciences, Department of Educational Sciences, Grote Rozenstraat 3, Groningen, 9712 TG The Netherlands

## Abstract

**Background:**

Integration of physical active academic lessons in the school curriculum may be an innovative way to improve academic outcomes. This study examined the effect of physically active academic lessons (Fit en Vaardig op school) on academic engagement of socially disadvantaged children and children without this disadvantage. In addition, the relationship between lesson time spent in moderate to vigorous physical activity and academic engagement was examined.

**Methods:**

From four elementary schools, 86 children who participated in the 22-weeks intervention were recruited (23 socially disadvantaged children). Academic engagement was determined by observing time-on-task during three classroom observation moments (start, midway and end observation). Every moment consisted of lesson observations after intervention lessons (post-intervention) and after regular classroom lessons (post-control). Differences in time-on-task between socially disadvantaged children and children without this disadvantage were analyzed using independent samples t-test. Differences between post-intervention and post-control observations were analyzed using multilevel analysis. Heart rate monitors measured the lesson time spent in moderate to vigorous physical activity. The relationship between percentage of moderate to vigorous physical activity during the intervention lessons and time-on-task was analyzed by calculation of partial correlations.

**Results:**

Time-on-task of socially disadvantaged children was lower than that of children without this disadvantage, differences were significant at the start post-control (t(65) = 2.39, p < 0.05) and post-intervention (t(71) = 2.75, p < 0.05) observation and at the midway post-control (t(68) = 2.45, p < 0.05) observation. Multilevel analysis showed that the time-on-task of all children was significantly higher during post-intervention in comparison with post-control lessons (ES = 0.41). No significant difference was found at the start observation, but there were significant differences at the midway (ES = 0.60) and end (ES = 0.59) observation. On average, the children were exercising in moderate to vigorous physical activity during 60% of de lesson time (14 minutes of an average lesson of 23 minutes). No significant relationships were found between percentage of moderate to vigorous physical activity during the intervention and time-on-task in the post-intervention lessons.

**Conclusions:**

Physically active academic lessons may positively influence time-on-task in children, which can contribute to academic success in the long term.

## Background

Children who experience risk factors in their home environment, such as low parental education levels and low family income, are more likely to have poor academic outcomes [1]. One of the predictors of academic outcomes is academic engagement in the classroom, which can be measured by the time spent focusing on academic tasks (time-on-task) [2,3]. Because academic engagement of children during a school day is positively related to academic success, it is important for schools to make effective use of official school time and ensure that children’s time-on-task is as high as possible [4]. Some studies showed that children from disadvantaged backgrounds have more difficulties with academic engagement. For example, children at-risk showed deficiencies in focusing on task in comparison with other children [5]. Interventions aimed at increasing academic engagement of children at-risk may contribute to the academic outcomes of these children [6].

A few studies showed positive effects of physically active academic lessons on time-on-task in subsequent regular classroom lessons. In one study effects were examined of short classroom-based physical activities (Energizers) that encourage children to move during academic instruction [7]. Third and fourth grade children showed a significant improvement in on-task behavior immediately after the Energizers. Another study explored the impact of the TAKE 10! program, in which movement and learning are integrated [8]. They found a reduction in off-task behavior of more than 20% after a physically active lesson and concluded that the active academic lessons had a positive effect on elementary school children’s behavior in class. Furthermore, the Texas I-CAN physically active academic lessons were found to prevent the reduction in time-on-task experienced after a period of inactivity in elementary school children [9]. In addition, positive effects of a three-year physically active academic intervention were found on academic achievement [10]. Although no time-on-task was measured in this study, the effects on academic achievement may, in part, be due to the effects on time-on-task.

To improve academic outcomes in children, physical activity of moderate to vigorous intensity (MVPA) during the lessons seems important [10,11]. This type of physical activity is linked with a substantial number of health benefits in children [12] and an increase of cerebral blood flow, which improves the oxygenation of the prefrontal cortex, a brain region that is important for cognitive functioning [13]. In addition, it stimulates immediate chemical changes in the brain, like increases in levels of dopamine and norepinephrine. These increased concentrations may immediately enhance attention and aid cognitive performance [14]. The question rises whether the lesson time spent in MVPA during physically active academic lessons influences the on-task behavior of the children immediately after the lessons. A study examined the short-term effects of moderate physical activity in children in an experimental setting. Primary school children walked on a treadmill at 60% of their estimated maximum heart rate for 20 minutes. After this exercise an improvement in attention and academic achievement was found [15]. Another study examined the time spent in the target heart rate zone (55%-80% of their maximum heart rate) and its relationship to cognitive tasks. No evidence was found that time within the target heart rate zone was related to cognitive performance. However, the results suggested that vigorous activities (time spent above the target heart rate zone) might have cognitive benefits [16]. Although this study suggested effects of vigorous activities, a review study showed that MVPA could facilitate cognition and that intense exercise could be detrimental to cognition [17].

Summarized, in the literature we found an increase in time-on-task after physically active academic lessons and physical activity of moderate to vigorous intensity seems to be an important prerequisite in order to find positive effects. This study focuses on socially disadvantaged children (SDC) in the Netherlands, based on parental education level, who academically underperform in comparison with children without this disadvantage (non-SDC) [18]. The first aim of the present study was to confirm that SDC were less on task during regular classroom lessons in comparison with non-SDC. The second aim was to investigate the effect of ‘Fit & Vaardig op school’ (F&V; Fit and academically proficient at school), physically active academic lessons, on the time-on-task of SDC and non-SDC immediately after F&V lessons. As it has been shown in the literature that disadvantaged children had poorer abilities to maintain their task attention [5], and that physical activity might be more effective for the attention of lower performers [19], it is hypothesized that SDC could benefit more from physically active academic lessons in comparison with non-SDC. The third aim was to examine if the lesson time spent in MVPA during the F&V lessons was related to the time-on-task in the lessons that immediately followed the F&V lessons. It was hypothesized that the more lesson time was spent in MVPA, the higher the time-on-task after the lesson. Highly intensive exercise was expected to be detrimental to cognitive performance [17].

## Methods

### Participants

As a part of the larger F&V intervention, 86 elementary children (23 SDC) from second or third grade classes of four elementary schools from the Northern Netherlands were recruited to participate in this study. All children participated in the F&V lessons. Any missing demographic information eliminated a child from all comparisons, resulting in a final sample of 81 children (mean age: 8.2 ± 0.65 years; 41 girls; 40 boys). Children were classified as SDC or non-SDC based on parental education, children of whom the person(s) responsible for the daily care completed less than three years of secondary school were classified as SDC. In the context of the Dutch educational policy schools are required to obtain this data from every child. Based on this data, schools receive extra funds for SDC in their student population [20]. The SDC (n = 20) and non-SDC (n = 61) classification data were obtained from the personal school files of the children. Table 1 shows the demographic information of the children, it can be found that there were significantly more SDC in grade 2 in comparison with grade 3 (p < 0.05). A subgroup of 67 children (mean age: 8.2 ± 0.59 years; 33 girls; 34 boys; 15 SDC) wore heart rate monitors during the F&V intervention. Written informed consent was obtained from the school principals of the participating schools. The parents/legal guardians were informed before the start of the intervention and were given the option to withdraw their permission for their child to participate at any time. Furthermore, the parents/legal guardians gave written informed consent for wearing heart rate monitors during the physical active academic intervention. All procedures were approved by the ethical committee of the Center for Human Movement Sciences of the University Medical Center Groningen/University of Groningen.

**Table 1:** **Participant demographics**

	**SDC (n = 20)**	**Non-SDC (n = 61)**	***p*** **value**
Age, years (sd)	8.3 (0.8)	8.2 (0.6)	^a^0.71
Gender, n boys (%)	10 (50.0)	30 (49.2)	^b^0.95
Second grade, n (%)	14 (70.0)	26 (42.6)	^b^0.03
BMI, kg/m^2^ (sd)	17.7 (3.4)	17.3 (3.0)	^a^0.61
Shuttle Run score (sd)	3.9 (1.6)	4.0 (1.6)	^a^0.78

^a^Independent t-test. ^b^Chi-square test.

### Intervention

The F&V intervention consisted of physical active mathematics and language lessons that were taught in the classroom. The main (long-term) goal of F&V was to improve academic skills and physical fitness of SDC and non-SDC. Children participated in the F&V program for 22 weeks, three times a week. During each lesson the children stood behind or beside their school desk. In each lesson 10–15 minutes were spent on mathematics and 10–15 minutes on language. The main focus was on reinforced concepts learned in earlier classes. The physical exercises were relatively easy to perform and aimed at exercising at moderate to vigorous intensity level. During the lessons all children performed basic exercises and specific exercises simultaneously. The specific exercises were performed when the children solved an academic task. For example, the word ‘dog’ must be spelled by jumping in place for every mentioned letter or the children had to jump 6 times to solve the multiplication ‘2x3’. Similar academic tasks with different words or sums were exercised during one lesson. The basic exercises were performed during the remaining part of the lesson (for example when the children were thinking about a sum). Basic exercises were for instance, marching, jogging or hopping in place. A more complete description of the intervention and its implementation has been published elsewhere [21].

### Design and procedure

In this study a within-subject design was used to determine whether physically active F&V lessons affected the time-on-task in the regular classroom lessons that immediately followed the F&V lessons. To do this, the time-on-task during a regular classroom lesson that followed a F&V lesson (post-intervention) was compared to the time-on-task during a regular classroom lesson that followed a regular inactive lesson (post-control). On every participating school post-intervention and post-control observations took place at the start, midway and the end of the 22-week F&V intervention period (see Table 2). In order to improve the reliability of the task-observations, at the start and midway observation two post-intervention and two post-control observations were performed on every school in one week. At the end observation, one post-intervention and one post-control observation was performed on every school in one week. On each school, the post-intervention and post-control lessons were observed at different schooldays at about the same time of day (always before lunch time). A post-intervention observation was followed by a post-control observation in the same week. All observations started when the regular classroom lesson, in which the children returned to their academic content, began. To assess the intensity of the F&V lessons, part of the children wore heart rate monitors during each physically active F&V lesson that was followed by observations.

**Table 2:** **Design of time-on-task (ToT) observations and heart rate measurements during the lessons**

**Start**		**Midway**		**End**	
**Lesson**	**ToT observation**	**Lesson**	**ToT observation**	**Lesson**	**ToT observation**
1.1 F&V lesson*	Post-intervention	2.1 F&V lesson*	Post-intervention	3.1 F&V lesson*	Post-intervention
1.2 Regular lesson	Post-control	2.2 Regular lesson	Post-control		
1.3 F&V lesson*	Post-intervention	2.3 F&V lesson*	Post-intervention	3.2 Regular lesson	Post-control
1.4 Regular lesson	Post-control	2.4 Regular lesson	Post-control		

^*^Heart rate measurements were performed.

### Time-on-task observations

The time-on-task of the children was observed through time sampling, by using a modified version of the observation method described by Grieco et al. [9]. Every child was observed for the duration of five seconds, before the observer moved on to the next child. To support the observer an audio file was played through a headphone with beeps with an interval of five seconds. When every child was observed once, the observers started with the first child again and repeated this sequence for the remaining part of the lesson. We aimed at observing each child 15 times a lesson (50 minutes of observations in an average class of 20 children), this criteria was not always met due to lesson interruptions (for example recess or lunch break). Eventually each child was observed 10–16 times a lesson. It was observed if a child showed on-task or off-task behavior. On-task behavior was defined as any behavior in which a child was attentive to the academic instruction or actively engaged in the appropriate task, as assigned by the teacher. All behaviors that were not on-task were noted as off-task behavior. Off-task behavior included fidget and listless behavior, for example wiggling on the chair, needless moving without paying attention, placing the head on the desk, talking to or looking at other children. During the start observation the inter-rater reliability of the observation method was determined. Five lessons were observed by two observers, the inter-rater reliability appeared to be of substantial agreement [22,23], κ = 0.73.

### Assessment of lesson intensity

Prior to the F&V intervention a maximal endurance test (20 m Shuttle Run test) was performed during a physical education class. This test is an item of the Eurofit and consisted of running back and forward over 20 meter at an increasing speed [24]. Heart rate data were collected using team heart rate monitors [25]. The heart rate monitors averaged and stored heart rate every second throughout the test time. The maximum heart rate (HRmax) was determined by visual inspection of the heart rate curve that was obtained from the test.

Heart rate data were also collected during the F&V lessons. Exercising with heart rates between 60% and 90% of HRmax was considered as MVPA. Exercising above 90% of HRmax was considered as highly intensive exercise [26,27]. From the heart rate data, the lesson time spent in MVPA was calculated for each child for every observed F&V lesson.

### Data analysis

The percentages of time-on-task were calculated for each child by dividing the number of observations in which the child performed on-task behavior, by the total number of observations during that lesson. Next, the two post-intervention and two post-control observations that were performed at the start were merged into one post-intervention and one post-control observation by calculating the mean time-on-task. The same merging took place for the midway observation. The time in certain heart rate zones was calculated (0-60%, 60-90% and 90-100% of HRmax) per child per lesson. By dividing the lesson time spent in 60-90% of HRmax with the total lesson time, the percentage of MVPA per child per F&V lesson was calculated. Pearson’s correlations were calculated between time-on-task, percentage of MVPA and participant demographics in order to detect important covariates. Demographics that significantly correlated with time-on-task or percentage of MVPA were used as covariates in further analyses.

Differences in time-on-task between SDC and non-SDC during post-control and post-intervention lessons (aim 1) were analyzed using independent samples t-tests (SPSS, version 20.0). Differences between post-intervention and post-control observations (aim 2) were analyzed using multilevel modeling (MLwiN 2.29). Time was nested within children and each individual had six observations. Multilevel models were calculated for the time-on-task of the children. The first model contained only the important covariates. Next, condition (post-intervention and post-control; model 2), the interaction between condition and SDC (model 3) and the interaction between condition and observation moment (model 4) were entered as possible predictors. Variables with a nonsignificant contribution to the model were removed from further analysis. Effect sizes were calculated as (mean post-intervention – mean post-control)/(pooled SD) [28,29].

The relationship between percentage of MVPA during F&V lessons and time-on-task was analyzed by calculation of partial correlations controlling for the effect of important covariates (SPSS, version 20.0). Statistical significance for all analyzes was set at 0.05.

## Results

### Descriptive statistics

Table 3 shows correlations between children’s time-on-task, MVPA and their demographic variables. No significant correlations were found with gender. Four significant correlations were found between time-on-task and grade, two between MVPA and grade. Three significant correlations were found between time-on-task and BMI, two between time-on-task and the Shuttle Run Test score and three between time-on-task and SDC. The correlations indicated that third grade children and children with a high score on the Shuttle Run Test were more on task and that SDC and children with high BMI were less on task. Third grade children spent more time in MVPA.

**Table 3:** **Correlations between children’s time-on-task (ToT), MVPA and participant demographics**

		**Gender**	**Grade**	**BMI**	**Shuttle run**	**SDC**
Start	ToT Post-Intervention	−0.04	0.43*	−0.08	0.07	−0.31*
	ToT Post-Control	−0.15	0.12	−0.01	0.00	−0.28*
	MVPA 1.1	0.08	0.32*	−0.01	0.08	−0.20
	MVPA 1.3	0.03	0.10	0.03	−0.10	−0.12
Midway	ToT Post-Intervention	−0.17	0.35*	−0.29*	0.30*	−0.16
	ToT Post Control	−0.15	0.18	−0.34*	0.22	−0.29*
	MVPA 2.1	0.04	−0.05	0.11	−0.20	−0.08
	MVPA 2.3	−0.02	0.15	0.09	−0.15	−0.05
End	ToT Post-Intervention	−0.04	0.26*	−0.33*	0.32*	−0.15
	ToT Post-Control	0.02	0.26*	−0.13	0.19	−0.13
	MVPA 3.1	−0.04	−0.38*	0.09	−0.21	−0.11

*Significant correlation (significance was set at 0.05).

### Time-on-task

#### Time-on-task differences between SDC and non-SDC

**Figure 1:**
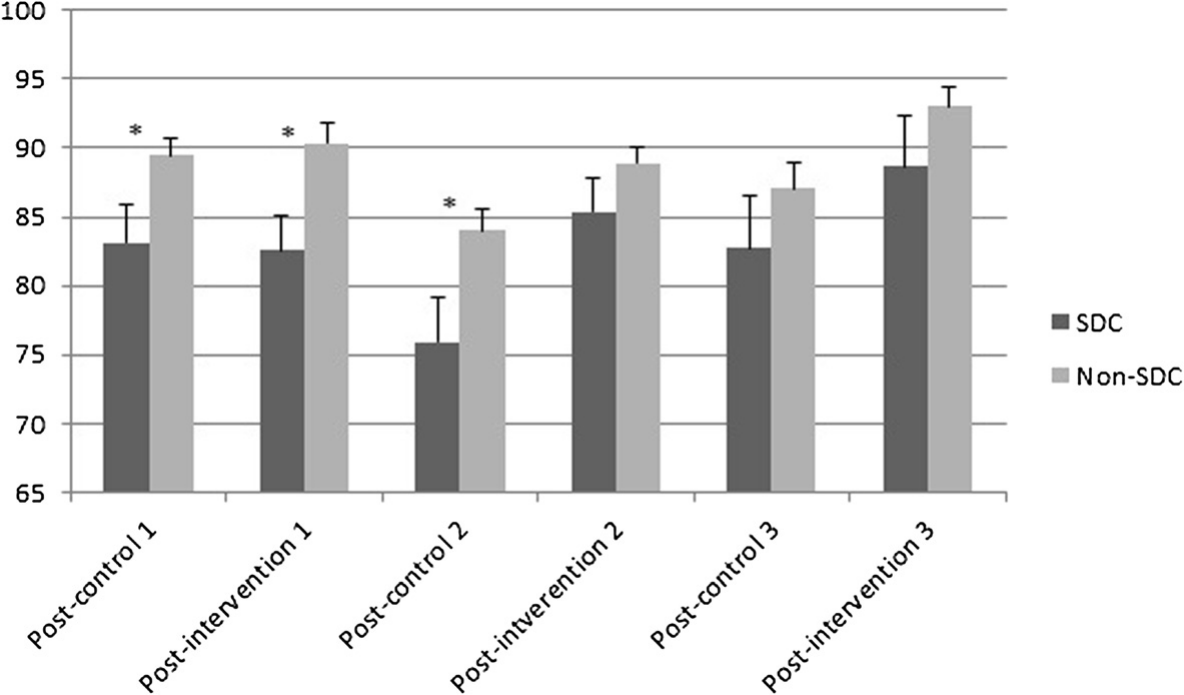
Mean percentages time-on-task with standard error bars of SDC and non-SDC during post-control and post-intervention lessons at start (1), midway (2) and end (3) observation. *Significant difference between SDC and non-SDC (p < 0.05).

Some children were absent during the start, midway and/or end observation. In case of absence during the lessons for more than 50 percent, children were excluded from all analyses. Common reasons for missed observations were absence from school or doing schoolwork outside the classroom. Figure 1 shows that the time-on-task of SDC was lower than that of non-SDC during the post-control and during post-intervention lessons. Differences were significant at the start post-control (t(65) = 2.39, p < 0.05) and post-intervention (t(71) = 2.75, p < 0.05) observation and at the midway post-control (t(68) = 2.45, p < 0.05) observation. No significant differences were found at the end observation.

#### Time-on-task differences between post-intervention and post-control

Table 4 shows the mean percentages of on-task behavior during intervention and control situations for SDC and non-SDC. The mean time-on-task of all children ranged from 88.1-92.1% during post-intervention lessons and 82.3-87.9% during post-control lessons.

**Table 4:** **Mean percentage time-on-task (SD); n during start, midway and end observation period**

	**Observation**	**SDC**	**Non-SDC**	**Total**
Start	Post-Intervention*	82.6 (11.1); 19	90.4 (10.6); 54	88.4 (11.2); 73
	Post-control*	83.1 (11.6); 17	89.5 (8.7); 50	87.9 (9.8); 67
Midway	Post-Intervention*	85.4 (10.3); 18	88.9 (9.1); 58	88.1 (9.4); 76
	Post-control*	75.9 (12.4); 15	84.0 (11.0); 55	82.3 (11.7); 70
End	Post-Intervention	88.7 (13.9); 15	93.0 (11.2); 57	92.1 (11.8); 72
	Post-control	82.7 (14.5); 15	87.1 (14.2); 61	86.2 (14.3); 76

*Time-on-task of the two merged observations was not significantly different.

Table 5 displays the results of the multilevel analysis predicting children’s time-on-task. The effect of the covariate Shuttle Run score was not significant (p = 0.33), so higher or lower fit children did not differ on their time-on-task, this variable was removed from further analysis. Model 1 shows a significant effect of the covariates grade, BMI, SDC and observation moment on children’s time-on-task. Children in third grade were more on task and SDC and children with high BMI were less on task. In addition, the on-task behavior of all children was lower at the midway observation moment in comparison with the start observation. The second model indicates a significant effect of condition (post-intervention or post-control) on time-on-task favoring the post-intervention condition (p < 0.05; ES = 0.41). This model was an improvement of the first model (Δχ^2^ (1) = 17.6, p < 0.05). Inserting the interaction between SDC and condition (model 3) did not significantly improve the model (Δχ^2^ (1) = 0.1, p = 0.75). As no significant interaction effect was found (the intervention did not affect SDC and non-SDC differently), the interaction was removed from the model. In the final model (model 4) the interaction between observation moment and condition was inserted. The model was significantly improved by this insertion (Δχ^2^ (2) = 7.2, p < 0.05) in comparison with the second model. No significant effect of condition was found during the start observation (ES = 0.04). The significant interaction effects between condition and midway observation and condition and end observation indicated a significant effect of condition during the midway (ES = 0.60) and end (ES = 0.59) observation compared with the start observation.

**Table 5:** **Multilevel regression coefficients (B) and Standard Error (SE) for each factor predicting time-on-task**

	**Model 1**			**Model 2**			**Model 3**			**Model 4**		
	***B***	***SE***	***p***	***B***	***SE***	***p***	***B***	***SE***	***p***	***B***	***SE***	***p***
*Fixed effects*												
Random intercept	96.18	5.39		97.37	4.18		97.45	4.19		99.21	4.24	
Grade^a^	4.98	1.34	0.00	4.97	1.34	0.00	4.97	1.34	0.00	5.02	1.34	0.00
BMI	−0.75	0.22	0.00	−0.75	0.23	0.00	−0.75	0.23	0.00	−0.74	0.23	0.00
SDC^b^	−4.47	1.58	0.00	−4.57	1.57	0.00	−4.95	1.99	0.01	−4.58	1.57	0.00
Midway observation^c^	−2.90	1.25	0.02	−2.87	1.22	0.02	−2.88	1.22	0.02	−5.81	1.74	0.00
End observation^c^	0.51	1.25	0.68	0.67	1.22	0.58	0.67	1.22	0.58	−2.17	1.71	0.11
Condition^d^				4.19	0.99	0.00	4.02	1.13	0.00	0.40	1.72	0.82
Condition*SDC							0.71	2.34	0.76			
Condition*midway										5.63	2.41	0.02
Condition*end										5.54	2.40	0.02
*Random effects*												
Variance between students	12.57	5.40	0.02	13.28	5.35	0.01	13.32	5.36	0.01	13.70	5.35	0.01
Variance within students	108.39	8.21	0.00	103.24	7.82	0.00	103.18	7.81	0.00	101.14	7.66	0.00
Deviance	3250.83			3233.27			3233.18			3225.25		

^a,b,c,d^ Respectively second grade, non-SDC, start observation and control condition were the reference categories.

### Lesson intensity

#### Time in MVPA and time-on-task

For the analyses of the association between time-on-task and time in MVPA, children who were absent during the Shuttle Run test were excluded. Table 6 shows that the number of children per observation moment ranged from 38 to 59. Mean percentages of MVPA during F&V lessons ranged from 47-62% in SDC and 51-68% in non-SDC. The standard deviations were relatively large. On average, the children were exercising in MVPA during 60% of de lesson time, which is in accordance with 14 minutes of MVPA during an average F&V lesson of 23 minutes. During on average 39% of the lesson time the children were exercising at a low intensity level (<60% of HRmax) and in on average 1% of the lesson time the children were exercising at a high intensity level (>90% of HRmax). In both SDC and non-SDC no significant correlations (while controlling for grade) were found between the percentage of MVPA during a F&V lesson and the time-on-task in the regular classroom lesson that immediately followed the F&V lesson (Table 6).

**Table 6:** **Mean percentages MVPA per observation moment (SD); n, and partial correlations of MVPA with time-on-task**

	**Start**		**Midway**		**End**
	**Lesson 1.1**	**Lesson 1.3**	Lesson 2.1*	**Lesson 2.3**	**Lesson 3.1**
SDC	55.3(28.1); 14	60.0(26.6); 15	56.6(33.3); 11	47.2(31.3); 14	61.7(31.9); 12
	r = 0.08	r = 0.09	r = 0.14	r = −0.01	r = 0.15
non-SDC	68.0(26.7); 44	67.3(26.1); 44	62.2(33.0); 27	51.1(34.3); 45	52.4(35.2); 42
	r = −0.03	r = 0.08	r = 0.11	r = 0.17	r = −0.15
Total	65.0(27.4); 58	65.5(26.2); 59	60.5(32.7); 38	50.2(33.4); 59	54.5(34.4); 54
	r = 0.03	r = 0.09	r = 0.11	r = 0.16	r = − 0.07

^*^Heart rate measurements of one school were missing because of technical errors.

As the children were hardly exercising at a high intensity level (>90% of HRmax), it was not possible to calculate correlations between the percentage of highly intensive exercise and time-on-task.

## Discussion

The present study showed significantly lower time-on-task in SDC compared with non-SDC during regular classroom lessons. Time-on-task of both SDC and non-SDC was significantly higher during post-intervention than post-control lessons, indicating that the time-on-task of both groups may have benefited from the F&V lessons. In both groups there was no significant relationship between the percentage of MVPA during F&V lessons and time-on-task in the regular classroom lessons that immediately followed the F&V lessons.

### Time-on-task

The results showed that during regular classroom lessons, the on-task behavior of SDC was significant lower than the on-task behavior of non-SDC. It is well known that SDC academically achieve less than non-SDC [18], but less is known about the discrepancy in on-task behavior. Given the discrepancy that was found in the current study, focusing on the on-task behavior of SDC might contribute positively to academic success in the long term. Previous research showed that physically active academic lessons positively influenced children’s time-on-task in regular classroom lessons that followed [7-9]. The present study demonstrated that the F&V intervention was beneficial to the on-task behavior of both SDC and non-SDC. The intervention lessons did not affect the on-task behavior of SDC and non-SDC differently. Future research should further investigate whether or not in the long term the academic performance of SDC and non-SDC also benefit from the F&V intervention.

After F&V lessons the time-on-task of all children was significantly higher than the time-on-task after regular control lessons. Only the start observation did not show a significant difference between post-intervention and post-control lessons. It is possible that during the first post-intervention observations, the children had to get used to the observers and, as a consequence, were less on-task. But the argument could also be used reversed. It is possible that the children were more on-task during the first control lessons because they saw the researchers for the first time.

In this study academic engagement was measured by observing children’s time-on-task. It can be discussed whether or not time-on-task observation is an adequate method for measuring academic engagement. According to Johnson et al. [30] academic engagement refers to, inter alia, making an effort to learn, completing homework, coming to class and being attentive in class. Although time-on-task observations do not cover all these components of academic engagement, several studies have demonstrated that time-on-task (as a measure of academic engagement) was positively related to academic achievement [3,31].

### Lesson intensity

In the current study we found that the children showed MVPA in on average 60% of the lesson time (in accordance with 14 minutes of an average 23 minutes F&V lesson). To our knowledge, this study was the first to assess the amount of MVPA of physically active academic lessons through heart rate measurements. Other studies used other instruments to assess MVPA, i.e. accelerometers [32,33] or indirect calorimeters [33], and also showed that the physical activity level of physically active academic lessons was mainly MVPA. The present study extends these findings by investigating the relationship between MVPA and time-on-task.

As the children were hardly exercising at a high intensity level it was not possible to draw any conclusions about highly intensive exercise. The results demonstrated that the percentage of time in MVPA during F&V lessons was not related to the time-on-task in the lessons that immediately followed the F&V lessons. These results were similar for both SDC and non-SDC. The standard deviations showed that differences between children in the amount of lesson time spent in MVPA were large. So, some children were exercising in MVPA during the whole lesson time, while others were exercising at lower intensity levels. Apparently, more lesson time spent in MVPA during F&V lessons does not automatically lead to a higher percentage of time-on-task in the subsequent regular classroom lesson. These findings are in accordance with a previous study, in which no significant relationship between the time spent in the target heart rate zone (55-80% of HRmax) and cognitive performance was found [16]. It could be possible that not only MVPA, but also the change of sedentary classroom time into another activity (F&V lesson) may facilitate the on-task behavior in the subsequent regular classroom lesson. This is in accordance with studies that investigated the effect of recess breaks with time for free play. During recess breaks some children were physically active while others were spending their time standing in small groups, talking. These studies found that children were more on task, less fidget and behaving better in class when they had a break [34,35].

Physically active academic classroom lessons might have additional benefits. For example, because the lessons generate mainly MVPA, positive effects on academic performances as well as physical fitness are expected [36]. Furthermore, Best [14] suggests that an interaction between aerobic physical activity and cognitive engagement may have a stronger effect on cognitive functioning. Moreover, the physically active academic lessons save time because the extra physical activity does not come at the expense of academic instruction. In order to further investigate the additional benefits of moderate-to-vigorous physically active academic lessons on cognitive functioning and physical fitness future research is necessary.

### Limitations

This study has some limitations. First, although a binary classification of socioeconomic status is commonly used in the literature [37], it may partly account for the limited effects found between SDC and non-SDC in the current study. The advantage of using the current classification is that it can be obtained from the personal school files of the children. Secondly, the missing heart rate data at one time point could have influenced the results. However, as the findings at this time point are similar to the findings at the other four time points, we assume that the results were hardly influenced. Third, the small sample sizes (of mainly the SDC group) could have influenced the results and conclusions. Future research with a larger sample size is warranted to examine whether or not the lower on-task behavior of SDC is consistent and to further investigate the influence of physical active academic lessons on the on-task behavior of SDC.

## Conclusions

The on-task behavior of socially disadvantaged children was lower than that of children without this disadvantage during regular classroom lessons. Physically active academic F&V lessons may positively contribute to the time-on-task of socially disadvantaged children as well as children without this disadvantage. The F&V lessons generated moderate to vigorous physical activity in on average 60% of the lesson time and may therefore be beneficial to children’s health. However, no significant relationship was demonstrated between the physical intensity of the F&V lessons and time-on-task in the subsequent regular classroom lessons. The findings suggest that physically active academic lessons may be an innovative way for teachers to increase children’s academic engagement and physical activity without losing time intended for academic learning.

## Abbreviations

MVPA: Moderate to vigorous physical activity
SDC: Socially disadvantaged children
non-SDC: Children without a social disadvantage
F&V: Fit & Vaardig op school (Fit and academically proficient at school)
HRmax: Maximum heart rate
